# Supplementary material for: Comparison of Atmospheric and Lithospheric Culturable Bacterial Communities from Two Dissimilar Active Volcanic Sites, Surtsey Island and Fimmvörðuháls Mountain in Iceland
Source: Microorganisms. 2023 Mar 6;11(3):665. doi: 10.3390/microorganisms11030665 (PMC10057085; doi:10.3390/microorganisms11030665)
Supplement: Supplementary file 1 [file microorganisms-11-00665-s001.zip › SupplementaryMaterial-Figure S1.pdf]

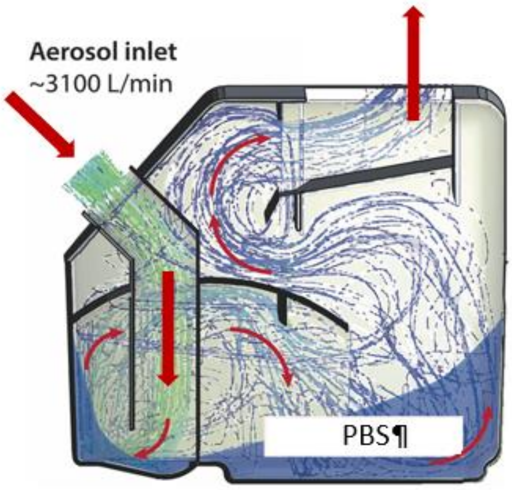


(**A**)

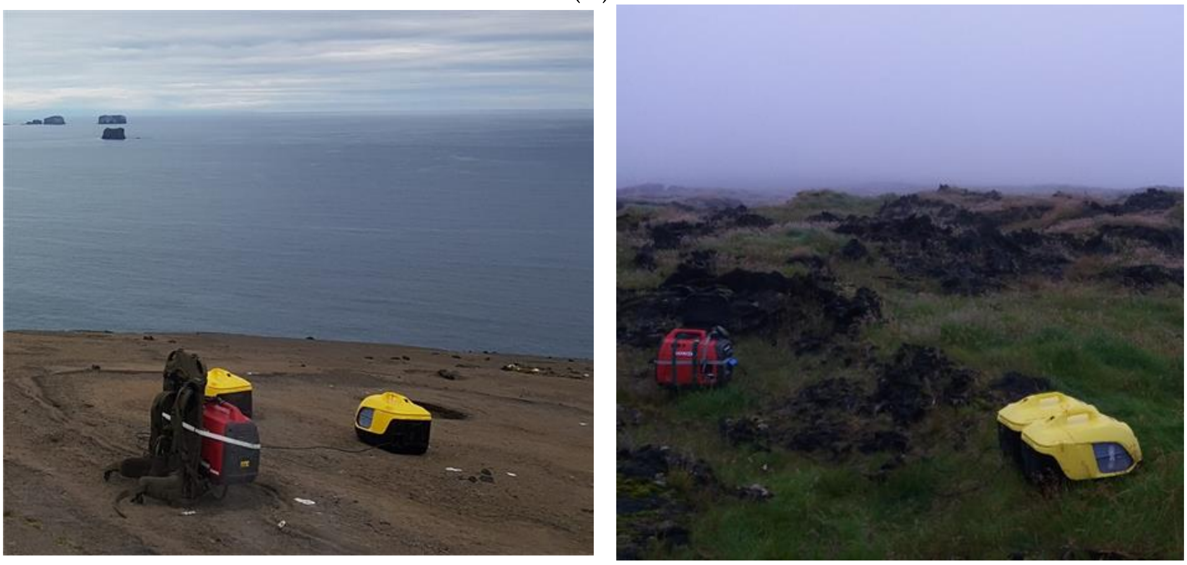

(**B**)

**Supplementary Material – Figure S1** (**A**) Schema of the high-flow rate impinger used in this study (modified from Šantl-Temkiv et al., 2017) and (**B**) pictures of it while in use in Surtsey (left picture: at the Viti station and right picture: at the Mávavarp station).
