# Supplementary material for: Comparison of Atmospheric and Lithospheric Culturable Bacterial Communities from Two Dissimilar Active Volcanic Sites, Surtsey Island and Fimmvörðuháls Mountain in Iceland
Source: Microorganisms. 2023 Mar 6;11(3):665. doi: 10.3390/microorganisms11030665 (PMC10057085; doi:10.3390/microorganisms11030665)
Supplement: Supplementary file 1 [file microorganisms-11-00665-s001.zip › SupplementaryMaterial-Figure S2.pdf]

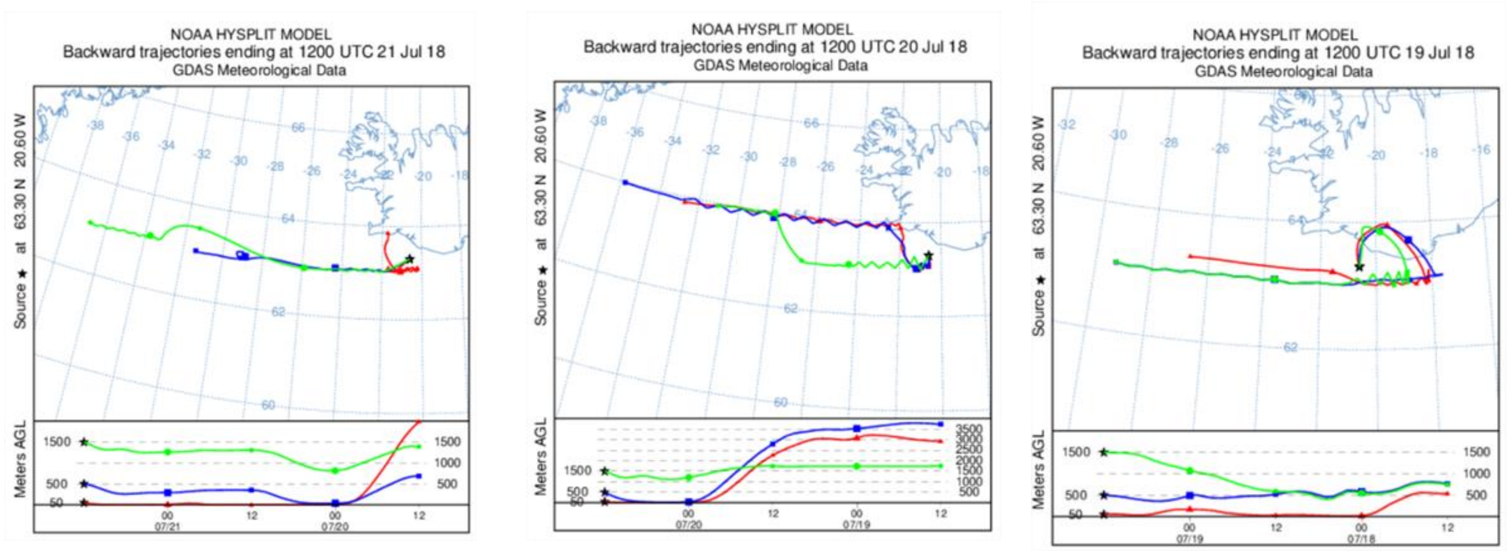

(**A**) Surtsey 2018

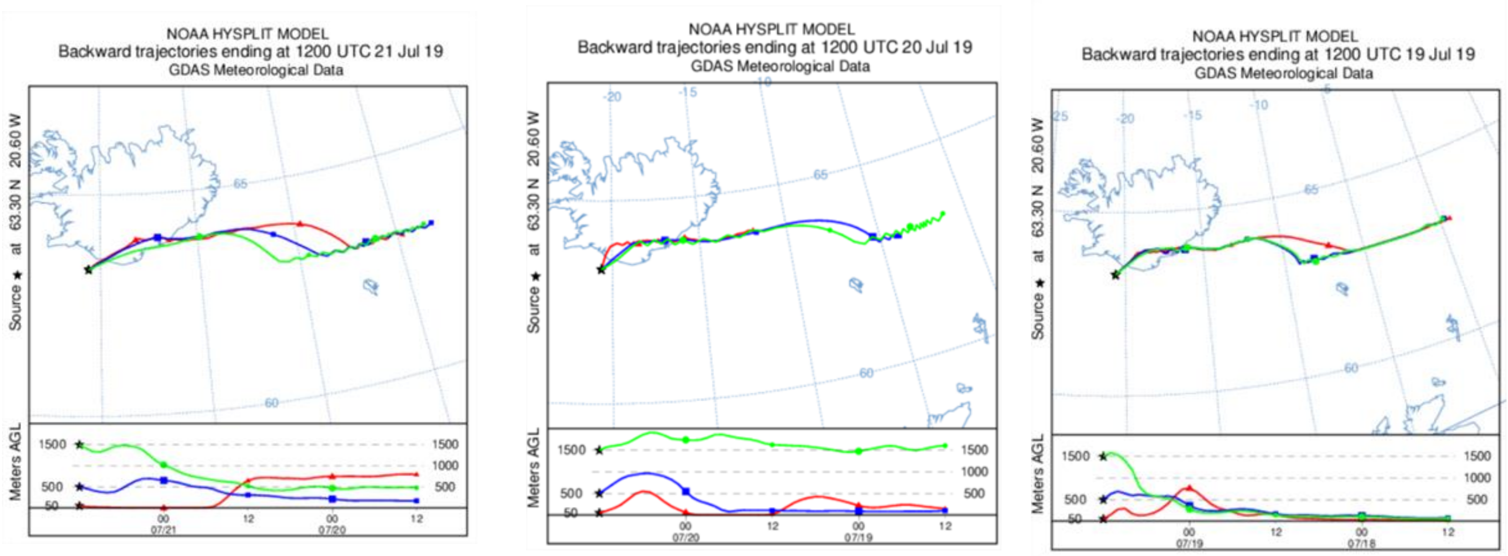

(**B**) Surtsey 2019

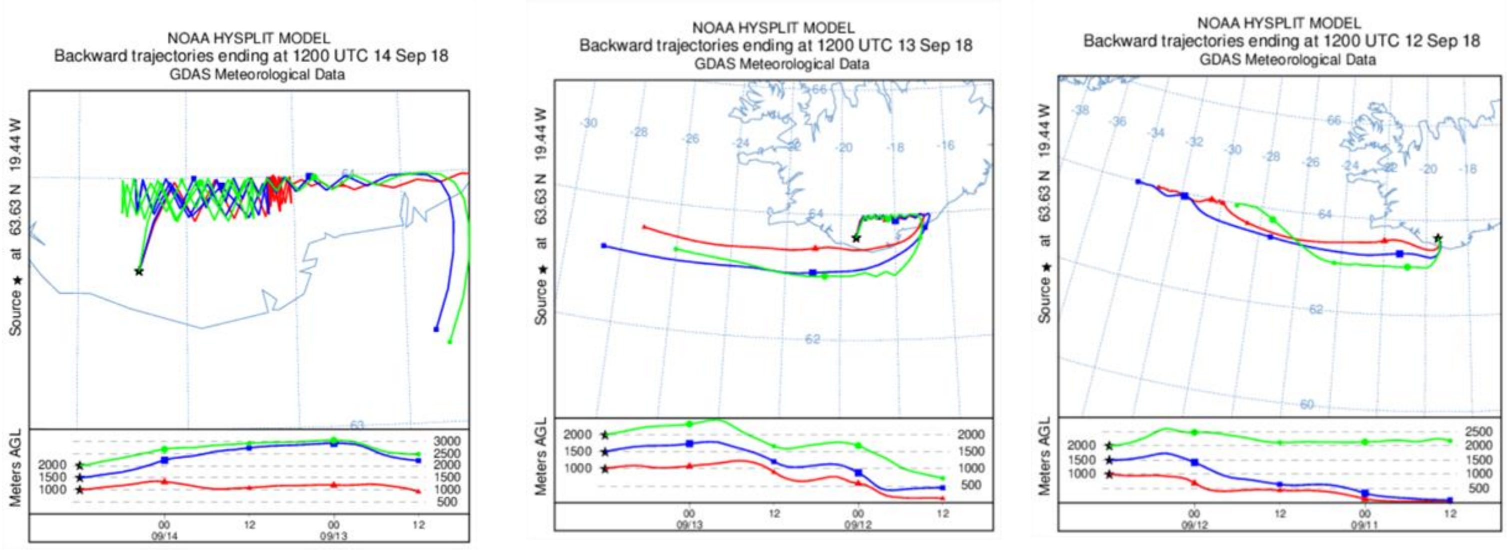

(**C**) Fimmvörðuháls 2018

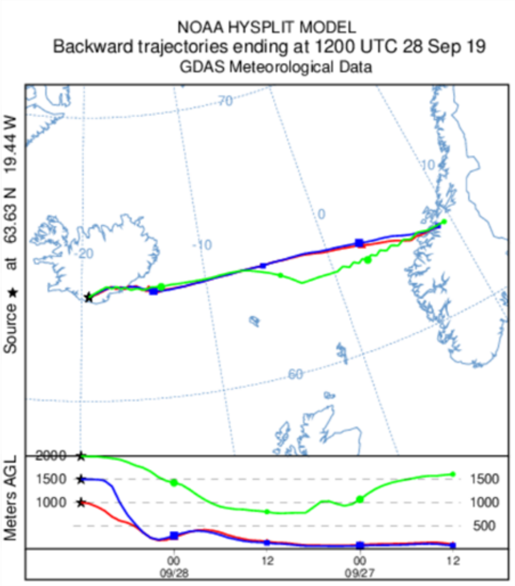

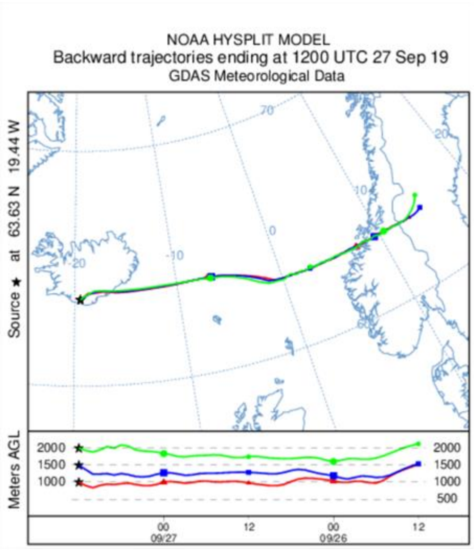

(**D**) Fimmvörðuháls 2019

Supplementary Material - Figure S2. 48-hours back trajectories computed by HYSPLIT for the samplings days at Surtsey in 2018 (A) and 2019 (**B**), and for Fimmvörðuháls in 2018 (**C**) and 2019 (**D**). The colors represent the air masses that ended at different altitudes at the sampling location and time. In Surtsey, green air masses ended at 1500m, blue air masses at 500m and red air masses at 50m whereas in Fimmvörðuháls, blue air masses ended at 1500m and red air masses ended at 1000m.
